# Major risk factors for obstructive sleep apnea monitored in the home. A cross-sectional study

**DOI:** 10.1590/1516-3180.2020.0689.R1.22042021

**Published:** 2021-10-29

**Authors:** Ricardo Silva, Tharcisio Pereira Brito, Antônio Cavalcanti Wanderley, Renata Botelho Frota, João Cateb Melo

**Affiliations:** I MD. Full Professor, Department of Cardiology, School of Medicine, Universidade Federal do Ceará (UFCE), Fortaleza (CE), Brazil.; II MD. Postgraduate Student, Department of Cardiology, School of Medicine, Universidade Federal do Ceará (UFC), Fortaleza (CE), Brazil.; III MD. Postgraduate Student, Department of Cardiology, School of Medicine, Universidade Federal do Ceará (UFC), Fortaleza (CE), Brazil.; IV MD. Attending Physician, Clínica Cateb Melo, Fortaleza (CE), Brazil.; V MD. Attending Physician, Clínica Cateb Melo, Fortaleza (CE), Brazil.

**Keywords:** Body mass index, Sleep apnea, obstructive, Hypertension, Age, Associated, Body density

## Abstract

**BACKGROUND:**

Obstructive sleep apnea (OSA) is characterized by recurrent pharyngeal wall collapse during sleep caused by anatomical or functional changes associated with obesity or dislocation of maxillofacial structures.

**OBJECTIVE:**

To determine the major risk factors for obstructive sleep apnea monitored in the home.

**DESIGN AND SETTING:**

Cross-sectional study conducted in a private clinic in Fortaleza (CE), Brazil.

**METHODS:**

Between 2015 and 2018, 427 patients were screened for OSA with home-based monitoring, yielding 374 positives. Information was collected on age, sex, body mass index (BMI), hypertension, diabetes (DM), dyslipidemia, coronary artery disease (CAD), arrhythmia, peripheral artery occlusive disease (PAOD), heart failure (HF) and lung disease. The home sleep apnea test result was then compared with the clinical diagnosis. Lastly, parameters identified as significant in the univariate analysis were subjected to multivariate logistic regression.

**RESULTS:**

Male sex predominated, although not significantly. OSA was associated with hypertension, DM, dyslipidemia, age and BMI. The risk of OSA being associated with these parameters was 2.195 (hypertension), 11.14 (DM), 2.044 (dyslipidemia) and 5.71 (BMI). The association was also significant for BMI categories (normal, overweight or obese). No significant association was observed for CAD, arrhythmia, PAOD, HF or lung disease. After multivariate logistic analysis, only age and BMI (and its categories) remained significant.

**CONCLUSION:**

OSA was associated with hypertension, DM, dyslipidemia, age and BMI in univariate analyses, but only with age and BMI (and its categories) in multivariate logistic analysis.

## INTRODUCTION

Obstructive sleep apnea is characterized by recurrent pharyngeal wall collapse during sleep, caused by anatomical or functional changes associated with obesity or dislocation of maxillofacial structures.^1^

This condition is highly prevalent in patients with cardiovascular disease. In fact, the syndrome is accompanied by hypoxia, oxidative stress, sympathetic activation and endothelial dysfunction, all of which are mediators of cardiovascular disease.^2^

Moreover, several authors in the literature have stated that ageing affects the severity of obstructive sleep apnea syndrome and the associated cardiovascular risk.^3^ The relationship between obstructive sleep apnea and cardiovascular disease involves the mechanisms of platelet activation and inflammation. Therefore, abnormalities may be observed in laboratory tests based on markers for platelet activation and inflammation, such as mean platelet volume, platelet-lymphocyte ratio, red blood cell distribution width and neutrophil-lymphocyte ratio.^4^

The incidence of obstructive sleep apnea is 14% in men and 5% in women. The condition is diagnosed through a sleep study, and the gold standard for this is polysomnography. Because very few clinics offer this type of examination, the vast majority of cases (80%-90%) will most likely never be formally diagnosed. As a cheaper alternative, home-based monitoring may be performed without the presence of a technician.^5^

There is evidence to suggest the existence of an association between obstructive sleep apnea and metabolic syndrome or diabetes.^6^ The pathophysiology is not well understood, but intermittent hypoxia is likely to play an important role.^7^ In addition to hypoxia, sleep fragmentation leads to activation of the sympathetic nervous system, the hypothalamic-pituitary-adrenal axis and pro-inflammatory pathways or oxidative stress.^8^ Both intermittent hypoxia and sleep fragmentation have been shown to be associated with several inflammatory biomarkers, thus leading to systemic inflammation.^9^

Among cardiovascular disorders, obstructive sleep apnea predisposes to arrhythmia, including atrial fibrillation, which is diagnosed in 6% of obstructive sleep apnea patients (20% if male). Greater prevalence of atrial fibrillation is positively associated with greater severity of obstructive sleep apnea.^10^ In association with obstructive sleep apnea, QT prolongation is a risk factor for severe arrhythmia.^11^ The fact that obstructive sleep apnea predisposes to coronary disease is supported by data from a systematic review of three trials, which showed that 12% of 5,067 obstructive sleep apnea patients had myocardial infarction or stroke or needed myocardial revascularization, with a fatality rate of 25% for cardiovascular events.^12^

Systemic arterial hypertension is also closely linked with obstructive sleep apnea, especially resistant systemic arterial hypertension. Among the drugs used to treat systemic arterial hypertension, angiotensin receptor blockers combined with continuous positive airway pressure can reduce pressure, while antimineralocorticoids moderately reduce the severity of obstructive sleep apnea.^13^ Continuous positive airway pressure is standard therapy in cases of severe obstructive sleep apnea. In a study monitoring 554 obstructive sleep apnea patients for cardiovascular outcomes, 50 cardiovascular events occurred in 44 patients over an average follow-up period of 5.9 years. Events were more frequent in patients with severe obstructive sleep apnea. The risk of cardiovascular events was 2.66 times greater in the group not treated with continuous positive airway pressure.^14^

The association between obstructive sleep apnea and cardiovascular disease has been tested in populations with different ethnic backgrounds. For example, the Australian Longitudinal Study on Male Health followed 13,423 men and found prevalences of 2.2% and 7.8% for men aged 18-25 and 45-55 years, respectively. Obstructive sleep apnea was significantly associated with age, unemployment, asthma, chronic obstructive pulmonary disease/bronchitis, diabetes, hypercholesterolemia, systemic arterial hypertension, heart failure, angina, depression, anxiety and schizophrenia.^15^ Obstructive sleep apnea is also highly prevalent among Iranians and, in one study, was associated with cardiovascular disease (26%) and systemic arterial hypertension (74%).^16^

## OBJECTIVE

To determine the major risk factors for obstructive sleep apnea monitored in the home.

## METHODS

Between April 2015 and April 2018, we screened 427 patients for obstructive sleep apnea through home-based monitoring. The result was positive for 374 and negative for 53 (Table 1).

**Table 1.** Clinical characteristics versus obstructive sleep apnea

		Obstructive sleep apnea			
		No		Yes	
		n	%	n	%
**Sex**	Female	22	41.5%	143	38.2%
	Male	31	58.5%	231	61.8%
**Hypertension**	Yes	19	35.8%	206	55.1%
	No	34	64.2%	168	44.9%
**Diabetes**	Yes	1	1.9%	66	17.6%
	No	52	98.1%	308	82.4%
**Dyslipidemia**	Yes	15	28.3%	167	44.7%
	No	38	71.7%	207	55.3%
**CAD**	Yes	4	7.5%	47	12.6%
	No	49	92.5%	327	87.4%
**Arrhythmia**	Yes	5	9.4%	69	18.4%
	No	48	90.6%	305	81.6%
**PAD**	Yes	4	7.5%	17	4.5%
	No	49	92.5%	357	95.5%
**HF**	Yes	2	3.8%	18	4.8%
	No	51	96.2%	356	95.2%
**COPD**	Yes	3	5.7%	8	2.1%
	No	50	94.3%	366	97.9%

CAD = coronary artery disease; PAD = peripheral arterial disease; HF = heart failure; COPD = chronic obstructive pulmonary disease.

We also collected information on sex, age, body mass index (BMI) and diagnoses of obstructive sleep apnea, diabetes, dyslipidemia, coronary disease, arrhythmia, peripheral artery occlusive disease, heart failure and lung disease. Lastly, we correlated each clinical diagnosis with the result from the respective home sleep apnea test (Table 1).

The study protocol was approved on February 7, 2018, by the research ethics committee of the local university hospital and was filed under #2.489.575.

### Statistical analysis

Quantitative variables were expressed as mean values ± standard deviation, while categorical variables were expressed as frequencies and prevalence, in order to test for associations between risk factors and the presence of obstructive sleep apnea. Group parameters were compared using the Mann-Whitney U test due to their non-normal distribution. Potential associations between categorical variables were analyzed using Pearson’s chi-square test and Fisher’s exact test.

All analyses were performed using the SPSS Statistical Package for the Social Sciences (SPSS) software, version 22.0 (Norman H. Nie, C. Hadlai (Tex) Hull and Dale H. Bent, Chicago, United States), and the R 3.3.1 software (Ross Ihaka and Robert Gentleman, Auckland, New Zealand). Parameters identified as significant in univariate analyses were subjected to multivariate logistic analysis. The level of statistical significance was set at 5% (P < 0.05).

## RESULTS

Male sex was predominant, although not significantly (Table 2). Presence of obstructive sleep apnea was associated with systemic arterial hypertension, diabetes, dyslipidemia, age and body mass index. The risk of obstructive sleep apnea being associated with systemic arterial hypertension was estimated as 2.195 (Table 3). The corresponding figures for diabetes, dyslipidemia and body mass index were, respectively, 11.14 (Table 4), 2.044 (Table 5) and 5.71 (Table 6). BMI categories (normal, overweight or obese) were also associated with obstructive sleep apnea. On the other hand, obstructive sleep apnea was not associated with coronary artery disease, arrhythmia, peripheral artery occlusive disease, heart failure or lung disease. Nearly all the coronary disease patients (49 out of 53; 92%) had a history of revascularization by means of surgery or stent implantation. When the associations between obstructive sleep apnea and the variables of systemic arterial hypertension, diabetes, dyslipidemia, age and body mass index were subjected to multivariate logistic regression, only age and body mass index (and its categories) remained significant (Table 7).

**Table 2.** Sex versus obstructive sleep apnea

		Obstructive sleep apnea		
		Yes	No	Total
**Female**	n	143	22	165
	%	38.2%	41.5%	38.6%
**Male**	n	231	31	262
	%	61.8%	58.5%	61.4%
**Total**	**n**	**374**	**53**	**427**
	**%**	**100.0%**	**100.0%**	**100.0%**

P = 0.647.

**Table 3.** Hypertension versus obstructive sleep apnea

			Obstructive sleep apnea		
			Yes	No	Total
**Hypertension**	**Yes**	n	206	19	225
		%	55.1%	35.8%	52.7%
	**No**	n	168	34	202
		%	44.9%	64.2%	47.3%
**Total**		**n**	**374**	**53**	**427**
		**%**	**100.0%**	**100.0%**	**100.0%**

P = 0.009; odds ratio = 2.194.

**Table 4.** Diabetes versus obstructive sleep apnea (OSA)

			Obstructive sleep apnea		
			Yes	No	Total
**Diabetes**	**Yes**	n	66	1	67
		%	17.6%	1.9%	15.7%
	**No**	n	308	52	360
		%	82.4%	98.1%	84.3%
**Total**		**n**	**374**	**53**	**427**
		**%**	**100.0%**	**100.0%**	**100.0%**

P = 0.003; odds ratio = 11.14.

**Table 5.** Dyslipidemia versus obstructive sleep apnea

			Obstructive sleep apnea		
			Yes	No	Total
**Dyslipidemia**	**Yes**	n	167	15	182
		%	44.7%	28.3%	42.6%
	**No**	n	207	38	245
		%	55.3%	71.7%	57.4%
**Total**		**n**	**374**	**53**	**427**
		**%**	**100.0%**	**100.0%**	**100.0%**

**Table 6.** Body mass index (BMI) range versus obstructive sleep apnea

		Frequency	Percentage
**BMI**	18-25	66	15.5
	25-30	163	38.2
	30-35	121	28.3
	> 35	65	15.2
	Total	415	97.2
**Absent**		12	2.8
**Total**		**427**	**100.0**

P = 0.000; odds ratio = 2.89 for BMI of 25 to 30, 5.71 for BMI from 30 to 35 and 4.85 for BMI > 35.

**Table 7.** Multivariate logistic regression

Clinical characteristics	OR (95% CI)		P
**Hypertension**	0.917	(0.451-1.864)	0.81
**Diabetes**	6.088	(0.8-46.326)	0.081
**Dyslipidemia**	1.453	(0.718-2.94)	0.299
**Age**	1.049	(1.026-1.073)	< 0.001
**Body mass index**			
18-25			-
25-30	2.554	(1.167-5.588)	**0.019**
30-35	7.683	(2.906-20.31)	**< 0.001**
> 35	6.652	(2.131-20.76)	**0001**

OR = odds ratio; CI = confidence interval.

## DISCUSSION

Obstructive sleep apnea is highly prevalent in patients with cardiovascular disease and occurs predominantly in males (14% versus 5%).^2^^,^^5^ Our sample consisted of patients with suspected obstructive sleep apnea; thus, unsurprisingly, 87% tested positive (374/427). While polysomnography is the gold standard for diagnosing obstructive sleep apnea, low-cost home-based monitoring yields reliable results and does not require the presence of a technician,^5^ which justifies the choice of this type of test for the present study.

Interesting rheological data on patients with obstructive sleep apnea syndrome have been reported. Patients with obstructive sleep apnea have elevated morning fibrinogen levels and higher plasma viscosity, which correlate positively with indices of sleep apnea severity. These changes in blood rheology are independent of cardiovascular risk factors.^17^

We found that occurrences of obstructive sleep apnea were associated with diabetes, like in many other studies.^5^^,^^6^ However, we did not find any association with arrhythmia, which is a frequently reported association. Furthermore, we did not find any evidence of the otherwise well-documented fact that obstructive sleep apnea predisposes to coronary artery disease,^12^ but the latter was unusually severe in our sample (49 out of 53 coronary artery disease patients had previously been revascularized through surgery or stent implantation).

Another frequently reported association, between systemic arterial hypertension and obstructive sleep apnea, was confirmed in the present study.^13^ Moreover, the estimated risk of this association was very high (2.195). Like in the Australian Longitudinal Study on Male Health,^15^ obstructive sleep apnea was significantly associated with hypercholesterolemia in the present study, with an estimated risk of 2.044. However, despite the significance of the associations between obstructive sleep apnea and the parameters of systemic arterial hypertension, diabetes, dyslipidemia, age and body mass index (and its categories), only the last two variables remained significant after multivariate logistic analysis.

## CONCLUSION

Occurrences of obstructive sleep apnea were associated with hypertension, diabetes, dyslipidemia, age and body mass index in univariate analyses, but only with age and body mass index (and its categories) in multivariate logistic analysis.
